# Pleocytosis in a patient with relapsing polychondritis accompanied by meningoencephalitis: a case report

**DOI:** 10.1186/s12883-018-1059-7

**Published:** 2018-04-25

**Authors:** Jie Cao, Min Zhang

**Affiliations:** Department of Neurology, Tongji Hospital, Tongji Medical College, Huazhong University of Science and Technology, No. 1095 Jiefang Avenue, Wuhan, 430030 China

**Keywords:** Cerebrospinal fluid, Meningoencephalitis, Relapsing polychondritis

## Abstract

**Background:**

Relapsing polychondritis (RP) is an uncommon immune-related disease with unknown causes. It is characterized by inflammation of cartilaginous or non-cartilaginous structures, such as the ears, nose, respiratory tract, eyes, and joints. Neurological involvement is rare in RP.

**Case presentation:**

We report a case of pleocytosis in a 64-year-old man diagnosed as having RP with meningoencephalitis. The patient’s condition markedly improved following methylprednisolone treatment.

**Conclusions:**

To our knowledge, this is the first report of recurrent pleocytosis in a patient with RP accompanied by meningoencephalitis. Steroid pulse therapy is effective in most cases, and early diagnosis is of importance.

## Background

Relapsing polychondritis (RP) is a rare immune-related disease that primarily, but not exclusively, affects cartilaginous structures, such as the ears, nose, and laryngotracheobronchial tree. RP eventually leads to the deformation of these structures [1]. This disease has a wide array of clinical manifestations that can mimic other unrelated diseases, and it has no specific laboratory indicators. An involvement of the central nervous system (CNS) is rare in RP and is often not considered by physicians, which may result in delayed treatment.

Here, we report the case of a patient with pleocytosis who was initially considered to have tuberculous meningitis (TBM). However, further exploration finally led to a diagnosis of RP with meningoencephalitis.

## Case presentation

**Fig. 1:**
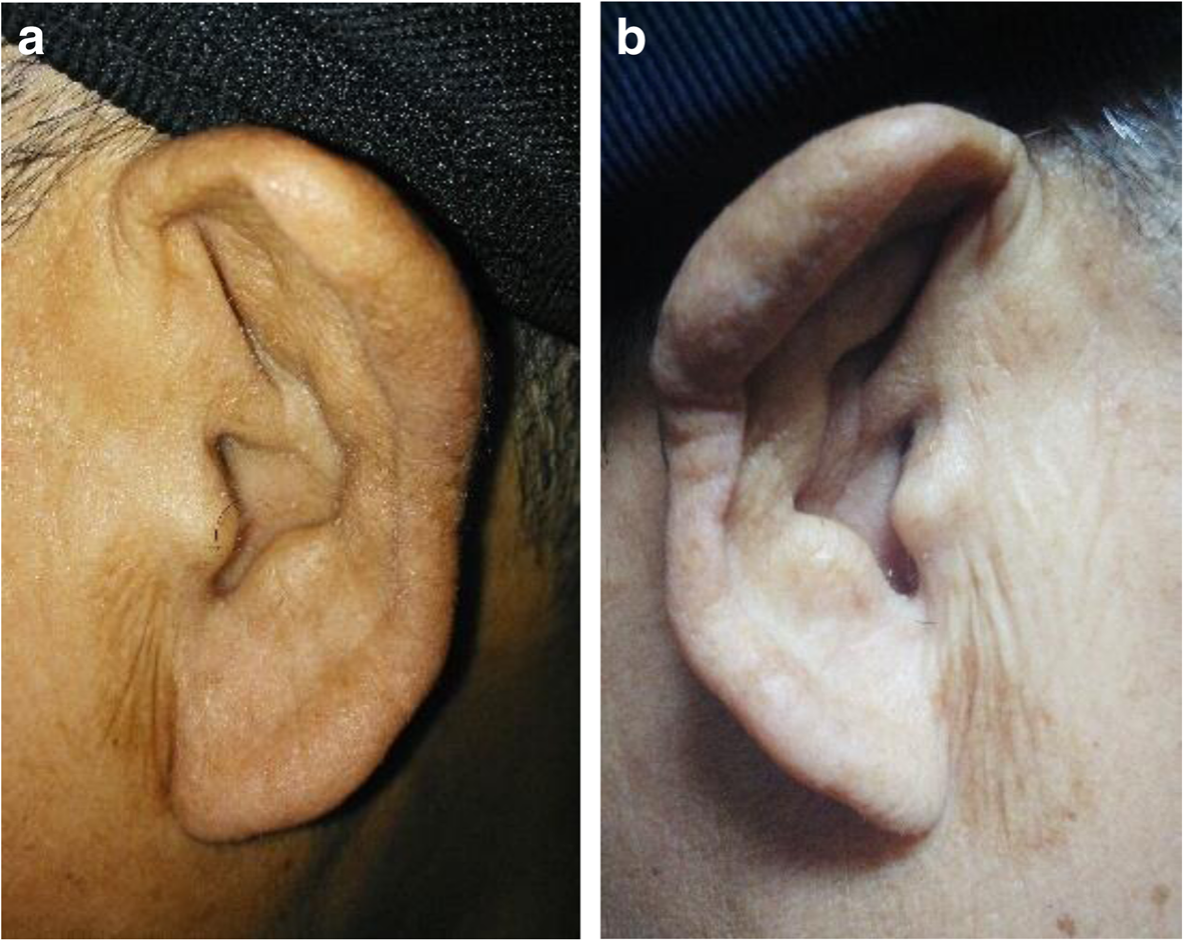
Bilateral swelling of the auricles in our patient. Swelling of left (**a**) and right (**b**) side of the auricles in our patient

A 64-year-old man with a history of headache for 4 months, dull responses, and gait disturbance for 3 months was admitted to our hospital. At admission, the patient had low-grade fever and replied very slowly (sometimes incorrectly) to questions. However, he was able to follow simple orders. The patient had symmetrical paralysis of both lower limbs, leaving him unable to walk without assistance. In addition, auricular swelling (Fig. 1) was observed upon physical examination. Bilateral Kernig’s sign was positive and ankle clonus was present. Assessment with the Mini-Mental State Examination (MMSE) yielded a score of 20/30, indicating impaired cognitive function. It was noteworthy that the patient had difficulty performing simple mathematical tasks as he had been an accountant for decades.

We reviewed the patient’s history and found that he had been in good health before the onset of symptoms. In particular, the patient had no history of trauma, smoking, alcohol abuse, allergy, recent vaccination, or hereditary factors. Headache and intermittent fever occurred after travelling, which led him to seek medical advice 4 months prior to his admission. At that time, TBM was suspected based on cerebrospinal fluid (CSF) tests, which revealed pleocytosis with a predominance of lymphocytes (Table 1). However, after a few weeks of antituberculous therapy, the patient developed several new symptoms, including ear swelling, tinnitus, dizziness, and gait disturbance.

**Table 1:** Cerebrospinal fluid profile of the patient

CSF profile				
Date	Protein (mg/dL) (normal range: 15-45)	Cell count (per mm^3^) (PMN/L/K)	Glucose (mmol/L) (normal range: 2.22-3.89)	Chlorides (mmol/L) (normal range: 120-132)
8/21/2016	77	52/208/260	2.74	117
9/3/2016	55	6/59/65	3.05	115
9/18/2016	66	−/−/8	3.66	117
9/28/2016	41	0/0/0	4.37	120
1/16/2017	43	−/−/42	3.79	117
2/4/2017	27	0/0/0	3.84	127

*CSF* cerebrospinal fluid, *PMN* polymorphonuclear cells, *L* lymphocytes, *K* karyocytes, −, no registry of cellular fraction

Subsequently, the patient was hospitalized three times with complaints of recurrent headaches, worsening gait disturbance, and other new symptoms including eyelid swelling and arthralgia. Two months after his first admission, the patient was diagnosed as having RP based on the Damiani and Levine criteria [2], and was treated with low-dose steroids. Antituberculous therapy was continued simultaneously. However, he showed little symptomatic improvement with treatment.

At the time of admission to our hospital, the patient’s CSF was assayed again. We detected pleocytosis (Table 1) and increased immunoglobulin G (IgG) levels (125 mg/L). CSF pathogen cultures and serologic tests for bacteria, viruses (such as human immunodeficiency virus, herpes simplex virus, Epstein-Barr virus, varicella-zoster virus, hepatitis viruses, and enteroviruses), fungi, parasites, and *Treponema* yielded negative results. The results of assays for tumor markers and paraneoplastic-related antibodies were also negative. In addition, assays for CNS vasculitis and autoimmune encephalitis yielded negative results.

Between the patient’s first clinical visit and admission to our hospital, his erythrocyte sedimentation rate (ESR) increased from 40 mm/h to 111 mm/h. An anti-nuclear antibody (ANA) test indicated a particle pattern with a titer of 1:100. The level of anti-cyclic citrullinated peptide antibody (anti-CCP) was 10 U/ml. The results of tests for other immune and rheumatoid biomarkers, such as anti-neutrophil cytoplasmic antibody, lupus anticoagulant, anti-phospholipid antibodies, and rheumatoid factors, were all negative or within the normal range. A Gene X-pert mycobacterium tuberculosis/rifampin detection test eliminated the possibility of a rifampin-resistant gene locus. Other indices were negative or within the normal ranges.

**Fig. 2:**
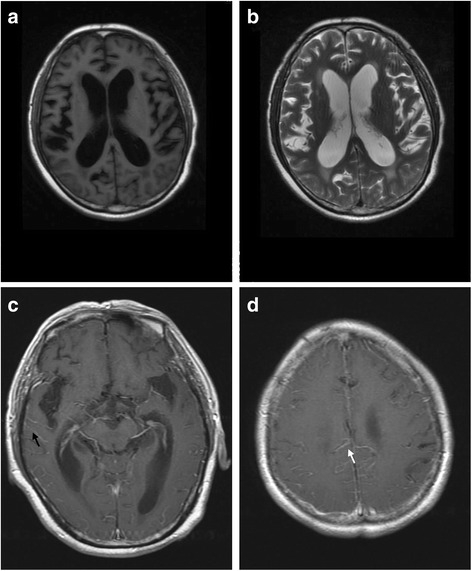
Head MRI of the patient. **a**, **b** T1- and T2-weighted images indicate supratentorial ventricular enlargement and abnormal signals in periventricular white matter, respectively. **c**, **d** Head MRI with gadolinium enhancement indicated leptomeningeal enhancement mainly located in the right temporal lobe (black arrow) and posterior cingulate gyri (white arrow)

Spinal magnetic resonance imaging (MRI) findings were normal. However, brain MRI indicated brain atrophy, hydrocephalus, and abnormal signals in periventricular white matter. Brain MRI with gadolinium enhancement showed leptomeningeal enhancement mainly in the right occipitotemporal and posterior cingulate gyri (Fig. 2), but not in the basal cisternal area, which is often indicative of typical tubercular meningitis.

Based on the aforementioned examinations, we suspected that the patient had RP with meningoencephalitis. Subsequently, intravenous methylprednisolone pulse therapy (500 mg for 3 days) with methotrexate (MTX, 12.5 mg, once per week) was administered. Supportive and symptomatic treatments were also provided. Three weeks after treatment initiation, the patient’s symptoms dramatically improved. We also observed a decrease in the ESR (20 mm/h), normalization of CSF assay results (Table 1), and an improved MMSE score of 28/30. Upon discharge, the patient had normal responses and was able to walk for 1000 m without help. No flare-up was reported at the 6-month follow-up visit.

## Discussion and conclusion

Initially, the patient was suspected to have TBM, which has a relatively high incidence rate in China. The detection of mycobacterium tuberculosis is necessary for a diagnosis of TBM. However, in reality, CSF cultures for mycobacterium tuberculosis have a low positive rate in patients with TBM [3]. Therefore, empirical treatment for TBM is usually initiated in patients clinically suspected of having the disease. Unfortunately, following several months’ treatment with standard antituberculous therapy, the patient’s condition did not improve but rather deteriorated, although we did not detect mutations in rifampin-resistant genes. In addition, a transient normalization of the lymphocyte count in the CSF during therapy and an atypical enhancement pattern on brain MRI aroused suspicions of a potential diagnosis of TBM. Moreover, the patient constantly complained of side effects of antituberculous therapy throughout the treatment course. Therefore, we cautiously stopped antituberculous treatment after a general discussion.

We considered a diagnosis of aseptic meningitis because repeated CSF gram stains and cultures were negative. Aseptic meningitis is a clinical syndrome characterized by meningeal inflammation not caused by pyogenic bacteria [4], but by some other factors such as infectious agents (e.g., viruses and parasites), neoplasms, anatomical structure-related events, vaccinations, drug-induced diseases, and autoimmune or inflammatory diseases. However, our patient likely did not have infectious aseptic meningitis because repeated CSF pathogen cultures and serologic tests were all negative. Negative results in tumor-related assays also indicated that the patient’s condition was unlikely to be due to the presence of neoplasms. Furthermore, there was no history of trauma or recent vaccination for the patient. Drug-induced aseptic meningitis was deemed unlikely in our patient, especially as he denied the use of any drugs before symptom onset.

After ruling out the above possibilities, the cause underlying the patient’s condition remained unknown. The signs and symptoms in our patient indicated the presence of both meningitis and encephalitis. To the best of our knowledge, prior to admission, the patient had only been diagnosed with RP, a rare autoimmune disease. We observed rheumatoid and immune-related abnormalities in our patient, such as elevated ESR, ANA, anti-CCP, and CSF IgG levels. Notably, CSF pleocytosis has previously been reported in patients with RP and, although rare, neurological involvement may be observed in RP [5]. Therefore, we suspected a correlation between RP and meningoencephalitis. Significant clinical improvement in our patient after treatment with a high-dose corticosteroid confirmed a diagnosis of RP with meningoencephalitis.

The etiology and pathophysiological mechanisms of RP remain largely unknown. The diagnostic criteria for RP are mainly clinical and no specific laboratory parameters have been confirmed to date. RP with meningitis or meningoencephalitis is rare, with approximately 3% of patients with RP having complications involving neurological symptoms [5].

Pleocytosis was detected in our patient. Vasculitis of the intracranial arteries, autoantibodies against the glutamate receptor GluRε2, and the presence of neutral glycosphingolipids in the CSF may explain CNS involvement in some cases of RP [6–8]. Although the pathogenesis of pleocytosis in RP is unclear, it seems to be associated with autoimmunity.

We observed a recurrent pattern of pleocytosis in 6 lumbar punctures from our patient. Karyocyte levels were normalized in the fourth CSF assay, which was conducted between adjacent assays with abnormalities before steroid treatment. To our knowledge, spontaneous normalization of karyocyte levels in CSF has not been previously reported in any other type of meningitis or meningoencephalitis. This may indicate a unique pattern of symptoms in this disease. Therefore, this is the first specific description of pleocytosis recurrence in a patient with RP. The marked normalization of karyocyte levels in CSF shortly after treatment may consequently invalidate previous diagnosis of TBM.

There are currently no randomized clinical trials of treatments for RP with meningitis or meningoencephalitis. Normally, empirical treatments are performed and steroid pulse therapy is preferred in most cases [7]. However, therapy may be ineffective or flare-ups may occur when an improper dosage is used or upon premature termination of therapy, as observed in our patient. The administration of immunosuppressive agents, such as azathioprine, MTX, cyclophosphamide, or cyclosporin A, may reduce the need for the long-term use of steroids and contribute to symptom control [9].

Meningoencephalitis is a rare complication of RP. Here, we report a case of RP with meningoencephalitis that was initially misdiagnosed as TBM. The patient recovered after steroid pulse therapy. This is also the first report of recurrent pleocytosis in a patient with RP accompanied by meningoencephalitis. Early diagnosis and steroid therapy are important in RP patients with meningoencephalitis. More case reviews and research are required to uncover the pathogenesis of RP and its rare neurological involvement.

## Consent

Informed consent was obtained from the patient for publication of this case report and any accompanying images.

## Abbreviations

ANA: Anti-nuclear antibody
Anti-CCP: Anti-cyclic citrullinated peptide antibody
CNS: Central nervous system
CSF: Cerebrospinal fluid
IgG: Immunoglobulin G
MMSE: Mini-Mental State Examination
MRI: Magnetic resonance imaging
MTX: Methotrexate
RP: Relapsing polychondritis
TBM: Tuberculous meningitis
